# Modeling the impact of vaccine campaigns on the epidemic transmission dynamics of chikungunya virus outbreaks

**DOI:** 10.1038/s41591-025-03684-w

**Published:** 2025-05-01

**Authors:** Pastor E. Pérez-Estigarribia, Gabriel Ribeiro dos Santos, Simon Cauchemez, Cynthia Vazquez, Ana Karina Ibarrola-Vannucci, Guillermo Sequera, Shirley Villalba, María José Ortega, Jose Luis Di Fabio, Danny Scarponi, Christinah Mukandavire, Arminder Deol, Águeda Cabello, Elsi Vargas, Cyntia Fernández, Liz León, Henrik Salje

**Affiliations:** 1 Laboratorio de Analisis y Modelado Basado en Datos (LAMBDA), Facultad Politécnica, Universidad Nacional de Asunción, San Lorenzo, Paraguay; 2 Facultad de Ciencias de la Salud, Universidad Sudamericana, Pedro Juan Caballero, Paraguay; 3 Department of Genetics, University of Cambridge, Cambridge, UK; 4 Department of Epidemiology of Microbial Diseases, Yale School of Public Health, New Haven, CT USA; 5 Mathematical Modelling of Infectious Diseases Unit, Institut Pasteur, Université Paris Cité, UMR 2000 CNRS, Paris, France; 6 Departamento de Virología, Laboratorio Central de Salud Pública, Asunción, Paraguay; 7 Unidad de Proyectos, Convenios e Investigación, SENEPA-Ministerio de Salud Pública y Bienestar Social, Asunción, Paraguay; 8 Cátedra de Salud Pública, Universidad Nacional de Asunción, Asunción, Paraguay; 9 Coalition for Epidemic Preparedness Innovations (CEPI), London, UK; 10 Dirección General de Vigilancia de la Salud, Ministerio de Salud Pública y Bienestar Social, Asunción, Paraguay; 11 Centro Nacional de Servicios de Sangre (CENSSA), Asunción, Paraguay

**Keywords:** Viral infection, Epidemiology

## Abstract

A licensed chikungunya vaccine now exists; however, it remains unclear whether it could be deployed during outbreaks to reduce the health burden. We used an epidemic in Paraguay as a case study. We conducted a seroprevalence study and used models to reconstruct epidemic transmission dynamics, providing a framework to assess the theoretical impact of a vaccine had it been available. We estimated that 33.0% (95% confidence interval (CI) 30.1–36.0%) of the population became infected during the outbreak. Of these individuals, 6.3% (95% CI 5.8–6.9%) were detected by the surveillance system, with a mean infection fatality ratio of 0.013% (95% CI 0.012–0.014%). A disease-blocking vaccine with 75% efficacy deployed in 40% of individuals aged ≥12 years over a 3-month period would have prevented 34,200 (95% CI 30,900–38,000) cases, representing 23% of all cases, and 73 (95% CI 66–81) deaths. If the vaccine also leads to infection blocking, 88% of cases would have been averted. These findings suggest that the vaccine is an important new tool to control outbreaks.

## Abstract (web summary)

A combination of seroprevalence analyses and modeling indicates that the availability of an infection-blocking CHIKV vaccine would have prevented 88% of cases during an outbreak.

## Main

Chikungunya virus (CHIKV) is an alphavirus transmitted by *Aedes* mosquitoes^1^. Outbreaks occur through global tropical and subtropical regions^2^. Infection in humans can cause a range of acute symptoms, including fever, headache and rash. In addition, there are long-term effects including severe arthralgia, which can take months to resolve^3^. Infection can also be deadly: an estimated 0.1% of cases detected by surveillance systems resulted in death, with older age, female sex and comorbidities linked to an increased risk of death^4,5^.

The first two chikungunya vaccines, IXCHIQ and VIMKUNYA, have now received approval by the Food and Drug Administration, the European Medicines Agency and other national regulatory agencies^6^. In anticipation of a vaccine, Gavi, which funds vaccines in lower-income countries, placed chikungunya vaccines on a learning agenda^7^. This means that there is currently insufficient evidence to fund the use of vaccines. Instead, it was felt that there needs to be a stronger evidence base to guide how to use a vaccine. As chikungunya outbreaks are often unpredictable in timing and duration, there remains interest in the development of vaccine stockpiles and vaccinating populations only once an outbreak is detected. Such an approach is used for cholera vaccines^8^. However, it remains unclear whether the dynamics of chikungunya epidemics would allow for such a deployment strategy. In particular, responding to an outbreak relies on early detection of the transmission through the surveillance system and then subsequent rollout of the vaccine.

Our understanding of chikungunya epidemiology relies largely on case data reported through surveillance systems. However, clinical misdiagnosis is common, and many infected individuals are asymptomatic or develop only mild symptoms. Further, as sick individuals may not visit formal healthcare providers, it is difficult to understand the relationship between the reported number of cases and the underlying patterns of transmission^9,10^. In this context, seroprevalence studies can be used to reconstruct the underlying infection patterns and quantify the probability that infected individuals are detected by the surveillance system^2,11^.

Here, we focus on a large nationwide CHIKV East–Central–South African (ECSA) lineage outbreak in Paraguay that occurred in 2022–2023, where 142,412 cases and 298 deaths were reported^12,13^. While there had been some cases reported in Paraguay each year before the outbreak, the largest number of annual cases previously had been 4,509 in 2015, with most years having fewer than 1,000 cases^14^. As we have a detailed understanding of the epidemiology, coupled with a new understanding of the underlying infection levels from a seroprevalence study, this outbreak provides an excellent opportunity to understand the impact of a vaccine had it been available at the time.

## Results

**Fig. 1:**
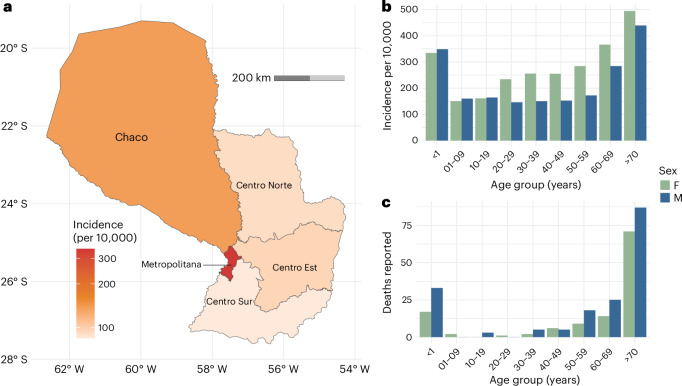
Reported case distribution. **a**, Map with the mean incidence within the five subregions. **b**, Incidence by age and sex of reported cases. **c**, Number of deaths by age and sex.

From September 2022 to September 2023, there were 142,412 chikungunya cases detected through the national surveillance system, with an average incidence of 208 cases per 10,000 people (2% clinical attack rate). The reported case incidence was the greatest in the capital region (Metropolitana, which contains Asunción) and the lowest in Centro Sur (Fig. 1a). The incidence of detected cases differed by age and sex, with a steady increase in incidence by age (Fig. 1b). The case incidence was 1.43 times higher in female than in male individuals. There was also a prominent pattern in deaths by age and sex (Fig. 1c): mortality cases were concentrated in the youngest (16.8% of deaths occurred in infants aged <1 year) and the oldest (53.0% of deaths occurred in those aged >70 years), and 122 and 176 deaths were in female and male individuals, respectively.

**Fig. 2:**
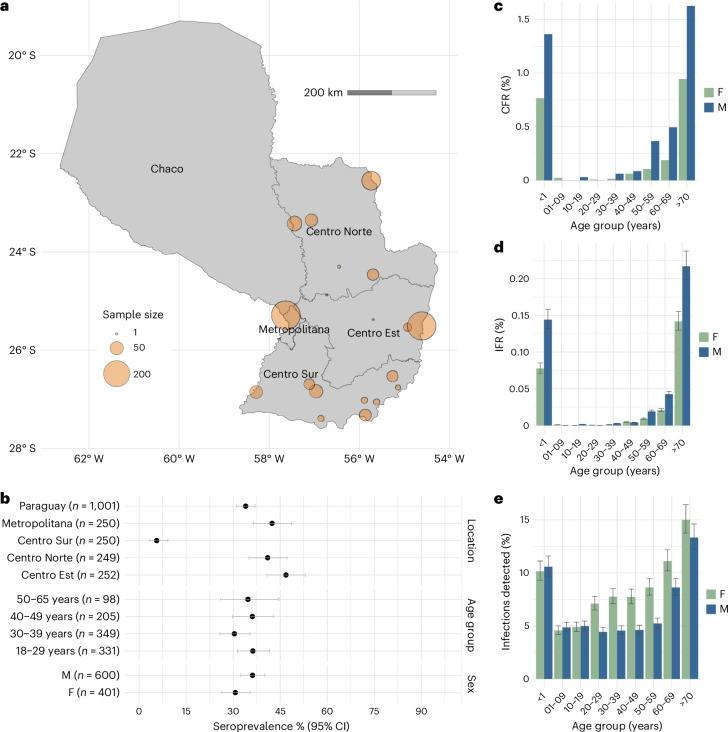
Seroprevalence study and underlying risk of severe disease. **a**, Location of samples by subregion. **b**, Seroprevalence by location, age and sex, with 95% CIs derived from a binomial distribution with varying sample sizes (*n*). **c**, CFR by age and sex. **d**, IFR by age and sex. **e**, Probability of severe disease (that is, probability of being detected by the surveillance system) by age and sex. The center points in **d** and **e** are derived from average seroprevalence values, and error bars are derived from the 95% CI of a binomial distribution (*n* = 1,001).

After the end of the outbreak, we obtained serum samples from 1,001 blood bank donors from across the country and tested them for immunoglobulin G (IgG) antibodies to CHIKV (Fig. 2a). We found that 340 of the 1,001 individuals (34.0%, 95% confidence interval (CI) 31.1–37.0%) were seropositive. Seropositivity was the greatest in the Centro Est region (47.2%, 95% CI 41.20–53.4%) and the lowest in the Centro Sur region (7.4%, 95% CI 4.8–11.3%) (Fig. 2b). Seropositivity did not differ significantly by age (*P* = 0.35) or sex (*P* = 0.08). After accounting for immunity before the outbreak, measured at 5% in the capital region from a prior seroprevalence study in 2017 (50 IgG-positive individuals from 1,000 tested) and assumed 0% elsewhere, and the distribution of the population across the country, we estimated a mean national attack rate of 33.0% (95% CI 30.1–36.0%), equivalent to 2.3 million (95% CI 2.1–2.5 million) infections. We estimated that the surveillance system detected 6.5% (95% CI 5.9–7.1%) of these infections, ranging from 11.3% (95% CI 7.40–17.6%) in Centro Sur to 2.0% (95% CI 1.8–2.4%) in the Centro Est region. The probability of an infected person being detected depended strongly on age and sex, ranging from 4.7% (95% CI 4.3–5.2%) for infected individuals who were 1–9 years old to 14.3% (95% CI 13.1–15.6%) for those aged >70 years (Fig. 2e and Extended Data Table 1). On average, infected female individuals were 1.32 times more likely to be detected than their male counterparts (95% CI 1.07–1.57 times). We estimated a mean case fatality ratio (CFR) of 0.21%, ranging from 0.004% in those aged 20–29 years to 1.23% in those >70 years of age. The very youngest individuals were also at a high risk of death, with those aged <1 year having a mean CFR of 1.08%. The average CFR was 0.29% in male individuals and 0.15% in female individuals (*P* value for the difference of <0.001) (Fig. 2). We estimated that the mean infection fatality ratio (IFR) was 0.013% (95% CI 0.012–0.014%), ranging from 0.00025% (95% CI 0.00023–0.00028%) in those aged 20–29 years to 0.18% (95% CI 0.16–0.29%) in those >70 years of age (Fig. 2d). When we assumed that the underlying infection attack rate differed by age and sex groups as per the seroprevalence study, we found that the IFR estimates were consistent to those obtained when we assumed equal exposure risk across groups (Extended Data Fig. 4).

To investigate the impact of unreported transmissions between 2017 and 2022, leading to greater immunity at the start of the outbreak, we ran a sensitivity analysis assuming that 10% of individuals in the Metropolitana subregion and 5% in the rest of the country were seropositive. This resulted in a slightly reduced mean national attack rate of 27.9% (95% CI 25.1–30.9%), a total number of infections of 1.92 million (95% CI 1.72–2.12 million) and a mean IFR of 0.016% (95% CI 0.014–0.017%) (Extended Data Table 2).

**Fig. 3:**
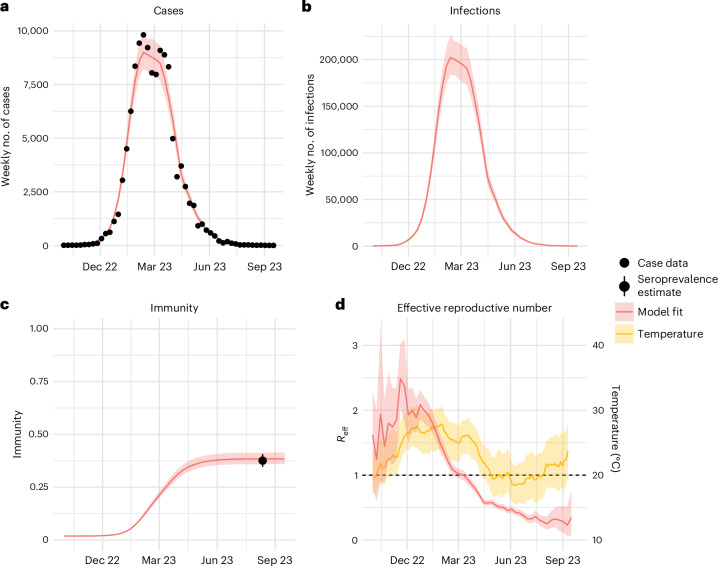
Mathematical model of the outbreak. **a**, Weekly number of reported cases. **b**, Weekly number of infections inferred from the model. **c**, Evolution of the nationwide levels of immunity inferred by the model. The error bar is derived from the 95% CI of a binomial distribution (*n* = 1,001). **d**, Inferred effective reproductive number (*R*_eff_) and average, minimum and maximum temperature. In **a**–**d**, model fit center lines are the median of the sampled posterior and model fit ribbons contain 95% of MCMC iterations.

We next built a transmission model that jointly estimated the changing transmission patterns during the outbreak and subsequent population immunity. Our model recovered the observed patterns of cases over time and the measured seropositivity (Fig. 3a,b). We found that immunity increased sharply to 28.6% (95% CI 26.5–30.9%) nationally 5 months into the epidemic and reached 34.8% (95% CI 32.0–37.7%) after 9 months (Fig. 3c). We found that the mean reproductive number over the first 3 months of the outbreak was 1.9 (95% CI 1.3–2.5) during the first weeks and remained above 1 for 22 weeks (Fig. 3d). Overall, 75% and 95% of infections occurred over a 29- and 35-week period, respectively. We found that the effective weekly reproductive number correlated with the country’s mean temperature in that week (Pearson correlation coefficient of 0.75).

**Fig. 4:**
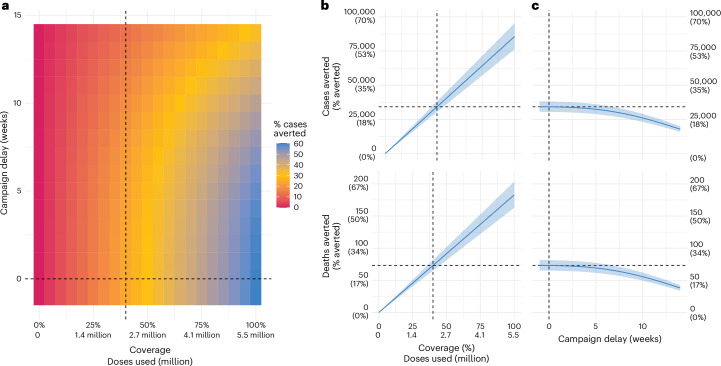
Results of the vaccine model. **a**, Proportion of cases averted for different values of coverage and delay. The dashed black line shows the base case scenario of 40% coverage with no delay between outbreak start and campaign vaccination. **b**, Cases (top row) and deaths (bottom row) averted when varying coverage and delay are fixed at 0 weeks. **c**, Cases (top row) and deaths (bottom row) averted when varying the delay between the detection of the outbreak and the start of the vaccine campaign, fixing coverage at 40%. In **b** and **c**, the ribbon represents propagated uncertainty from the epidemic model fit, where the vaccine impact is estimated using lower and upper epidemic parameter values.

**Extended Data Fig. 1:**
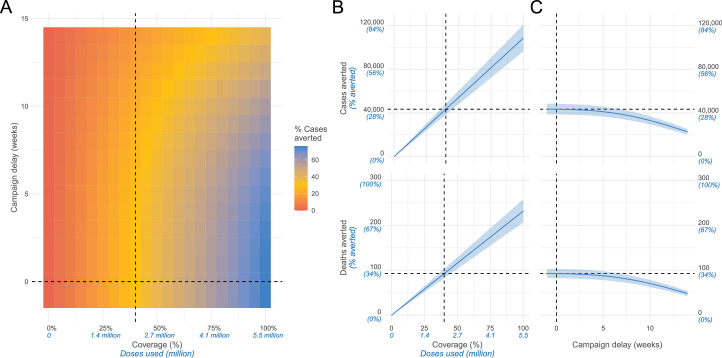
Results of vaccine model - sensitivity with a 98% vaccine efficacy. (**a**) Proportion of cases averted for different values of coverage and delay. The dashed black line shows the base case scenario of 40% coverage with no delay between outbreak start and campaign vaccination. (**b**) cases (top row) and deaths (bottom row) averted when varying coverage and delay are fixed at 0 weeks. (**c**) cases (top row) and deaths (bottom row) averted when varying the delay between the detection of the outbreak and the start of the vaccine campaign, fixing coverage at 40%.

Using our new understanding of the changing underlying transmission dynamics of the virus over the course of the outbreak, we explored the potential impact of a reactive vaccine campaign. To do this, we incorporated an additional compartment into the transmission model for vaccinated individuals, assuming that the vaccine had 75% efficacy against disease (but did not protect individuals against infection). We then used the fitted transmission parameters to rerun the epidemic but assumed that once the Ministry of Health reported the outbreak (week of 3 October 2022), a vaccine campaign was initiated in individuals aged 12 years and older (based on likely initial guidance for the vaccine) and that 40% of the population became vaccinated over a 3-month period. We used 40% as a benchmark and the rate of rollout based on expert opinion and the approximate levels of dengue vaccine (QDenga) uptake during the dengue epidemic in Brazil in 2024. We found that this deployment strategy would have required 2.2 million doses and averted 34,200 (95% CI 30,500–38,100) cases, including 17,100 (95% CI 15,500–19,000) cases with chronic sequelae and 73 (95% CI 65–81) deaths (Fig. 4a and Extended Data Table 3). This is equivalent to 156 cases, 78 chronic cases and 0.33 deaths averted per 10,000 doses, representing 23% of the cases and deaths that occurred. If, by contrast, only 20% of the population had become vaccinated, then the campaign would have averted 11% of the cases and deaths that occurred (Fig. 4b). In a scenario in which vaccine deployment was only initiated 3 months after the outbreak was detected, a campaign with 40% coverage would have averted 13% of the cases and deaths that occurred (Fig. 4c). Assuming a higher vaccine efficacy of 98% results in 31% deaths averted (198 cases and 0.42 deaths averted per 10,000 doses used) (Extended Data Fig. 1).

**Extended Data Fig. 2:**
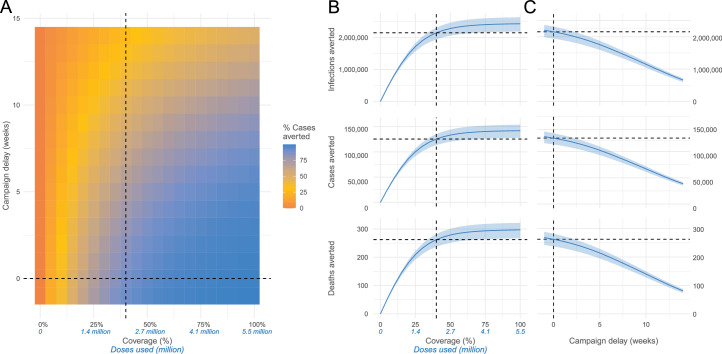
Results of vaccine model - sensitivity with infection-blocking vaccine. (**a**) Proportion of cases averted for different values of coverage and delay. The dashed black line shows the base case scenario of 40% coverage with no delay between outbreak start and campaign vaccination. (**b**) Infections (top row), cases (middle row) and deaths (bottom row) averted when varying coverage and delay are fixed at 0 weeks. (**c**) Infections (top row), cases (middle row) and deaths (bottom row) averted when varying the delay between the detection of the outbreak and the start of the vaccine campaign, fixing coverage at 40%.

From these initial results, we cautiously assumed that the vaccine only prevents disease, but vaccinated individuals can still become infected. To assess the impact of the vaccine if it also blocks onward transmission, consistent with what we believe about other live attenuated vaccines and the observed relationship between titers and the risk of infection and disease in chikungunya cohorts, we amended our model to prevent onward spread from infected vaccinated individuals, keeping everything else unchanged (Extended Data Fig. 2)^15^. We found that a 40% coverage in the same population with 75% efficacy against both infection and disease would have a substantial impact on the epidemic, with 88% deaths averted (573 cases and 1.20 deaths averted per 10,000 doses used).

## Discussion

We have used a large CHIKV outbreak in Paraguay to quantify the underlying detection probabilities of surveillance systems for chikungunya, the attack rate of the epidemic and the potential impact of a reactive vaccine campaign. We found that the vaccine could significantly reduce the burden of the virus, highlighting the important potential of this new technology.

Our findings provide an avenue for how to implement this new vaccine. Chikungunya epidemiology remains highly unpredictable in most settings. While some countries have evidence of endemic transmission at the national level, most locations experience infrequent outbreaks, and there may be a low inclination to initiate vaccine campaigns when the near-term burden remains highly uncertain and the durability of protection remains unclear^5,16^. By demonstrating that the vaccine can be used in a reactive manner, our study provides an evidence base for the establishment of stockpiles that can be readily deployed once an outbreak is detected. Overall, the outbreak lasted around 8 months beyond its declaration, with the reduction in the force of infection probably linked to climatic conditions coupled with increased population immunity.

This study highlights how sensitive surveillance systems are critical for an effective infectious disease response and should be prioritized for investment. In particular, reactive vaccine campaigns rely on surveillance systems detecting new outbreaks quickly. Due to the similarity in symptoms with dengue and other pathogens and limited access to testing, entire CHIKV epidemics can be missed^11,17^. However, in some countries within South America such as Brazil and Paraguay and potentially elsewhere, which only experienced their first outbreaks in 2014, surveillance systems seem to be better equipped to detect CHIKV outbreaks. The Ministry of Health declared an outbreak in October 2022, when only approximately 30 cases had been detected. The threshold for the optimal number of cases that should be used to trigger a vaccine response is unclear and requires careful investigation as, ultimately, there is a trade-off between initiating timely and appropriate responses to outbreaks and limiting false alarms. It is likely that the optimal threshold will be specific to the setting and will depend on surveillance and response capabilities.

The accelerated pathway to Food and Drug Administration approval has meant that we have a product that is close to being used. However, the absence of traditional phase 3 trials means that we continue to have a limited understanding of the efficacy of the vaccine. However, antibody data from studies of the vaccine are promising, with high, sustained titers in vaccinated individuals^15,18^. We would also expect live attenuated viruses to act much like a natural infection and, therefore, result in infection blocking. Hence, our sensitivity analysis showing a much larger impact than those based on disease blocking only may be reasonable.

While the major focus of chikungunya vaccines remains averting morbidity, especially long-term chronic sequelae resulting from CHIKV infection^2^, there is a growing realization that chikungunya is a deadly disease^4^. Nearly 300 individuals died during the Paraguay outbreak, with a mean CFR of 0.21%, which is higher than previous estimates from the Americas (ranging between 0.05% and 0.13%)^19^. The reasons for the higher CFR are unclear. It seems unlikely that viral factors are linked to the increased fatality as the same ECSA strain has been found elsewhere in the Americas. Instead, the higher-than-expected CFR may be linked to reduced case detection during this large outbreak, with only approximately 1 in 20 infections ultimately detected, potentially owing to the swamping of the surveillance system. We estimated an IFR of ~0.01%. Unlike CFRs, IFR estimates are robust to underlying biases in case surveillance. We are not aware of other studies that have directly quantified the underlying CHIKV IFR as the underlying level of infection has been rarely estimated. While the risk of death is the greatest among older adults, which can be directly protected by the vaccine model, there is also an elevated risk of death in the very youngest individuals, who remain currently ineligible for vaccination. However, assuming that the vaccine also blocks infection would provide indirect protection for this vulnerable population.

Our study also offers insights into the underlying disease patterns in infected populations. We found a strong effect of age on the probability of detection by the surveillance system. This is consistent with other reports of increasing probability of symptoms by age following infection^5^. We also found a higher probability of case detection in infected female individuals compared to male individuals. This pattern has been observed elsewhere, including in Brazil and Bangladesh^5,20,21^. Earlier work had suggested that the higher detection of cases in female individuals may be linked to underlying differences in risk behavior, with female individuals more likely to be in and around the home, where *Aedes* mosquitoes are typically found. However, our seroprevalence data suggest that an underlying difference in infection risk was not the main factor driving differences by sex in observed cases; instead, the data indicate an increased risk of symptoms in female individuals compared to their male counterparts following infection, consistent with the results from a prior seroprevalence study in Brazil^22^. Differential healthcare-seeking behaviors between male and female individuals may also contribute to this observation. By contrast, we found that the probability of death was significantly higher in male than female populations. These complex differences in the development of symptoms and the risk of severe disease between the sexes are potentially linked to complex immunological factors, consistent with those found across different infectious diseases^23^. Given the prominent patterns of symptoms and risk of death by age, older adults should be prioritized for vaccination.

Our study has some limitations. We used serum samples from blood banks in our seroprevalence study, which means that our study included only adults and we had limited data on participants. Our seroprevalence study was also not powered to investigate differences in infection risk by age and sex. Larger studies may identify more subtle differences by sex in some age groups. Individuals who donate blood may have a different underlying risk of chikungunya than the rest of the population; however, given the high attack rate in the country, any effect is likely to be minimal. Our seroprevalence study may also be affected by false positives due to cross-reactivity with the Mayaro virus. However, we expect that it would have only a minimal impact on the estimated overall number of CHIKV-infected individuals as the vector that transmits Mayaro is mostly found in sparsely populated forested regions where the *Haemagogus* mosquito resides^21^. The applicability of our results to other regions where the CHIKV circulates is unclear, especially as the outbreak was detected relatively quickly in Paraguay. Finally, the outbreak in Paraguay was large, with approximately 3 in 10 individuals infected. The impact of the vaccine in smaller outbreaks may not be as significant. Nevertheless, the largest outbreaks are ultimately the most disruptive to public health and general healthcare provision. Therefore, a significant reduction in the impact of these major epidemics is a major step forward in our battle against the virus.

With explosive outbreaks, CHIKV remains a threat to public health across large areas of the globe. However, we now have a tool to combat these outbreaks and, following this work, an evidence base to support the rapid deployment of the vaccine after outbreak detection. The development of appropriate regional stockpiles, robust vaccine deployment protocols and local vaccine licensure will be the necessary next steps in using the vaccine.

## Methods

### Ethical approval

The seroprevalence study was conducted as part of the Paraguay Ministry of Health’s public health emergency response and, therefore, did not require ethical approval. The seroprevalence study has not been published (either in full or in part) elsewhere. Following a standard operating process, the Centro Nacional de Servicios de Sangre (CENSSA), with the signed authorization of the responsible director, provided all the donated blood samples used in this study. All blood samples were collected anonymously, with no identifiers that could link the sample to the original patient; there was no enrollment procedure, and the patient consent form was the standard used by the CENSSA. The biochemical analyses were conducted at the Central Public Health Laboratory of the Ministry of Health in Paraguay. The use of epidemiological surveillance data was authorized by the signature of the director of the DGVS (Dirección General de Vigilancia de la Salud). The mathematical modeling work was conducted on aggregate data and, therefore, did not constitute personally identifiable information.

### Epidemic case data

We used weekly case data from September 2022 to September 2023. All suspected chikungunya cases were reported to the DGVS, Ministerio de Salud Pública y Bienestar Social de Paraguay. Approximately 60% of suspected cases undergo confirmatory testing through PCR, with an additional 23% tested using IgM ELISA. Suspected infection cases are defined as persons with sudden onset of fever and arthralgia or disabling arthritis unexplained by another medical condition. Probable infections are defined as any suspected case with a positive laboratory result for CHIKV (IgM ELISA) or an epidemiological link to a confirmed case. Confirmed infections are suspected or probable CHIKV cases with positive results in real-time reverse transcription followed by PCR or viral isolation tests^12,24^.

### New seroprevalence study

We conducted a seroprevalence study using blood bank serum samples in four of the five subregions (defined by the Ministry of Health, ‘Ejes’) of Paraguay (Metropolitana, Centro Sur, Centro Este and Centro Norte). We were not able to obtain serum samples from the final subregion (Chaco). In each subregion, we worked with the local blood collection service. Samples were collected from 25 July 2023 to 23 August 2023 from persons aged 18–65 years attending a blood donation service, with a target sample size of 250 persons per axis. We used IgG Euroimmun ELISA kits to test the samples for evidence of IgG antibodies to CHIKV. Testing was conducted in the Laboratorio Central de Salud Pública, Paraguay. We adjusted our seroprevalence estimates to account for the 98.6% sensitivity and 98% specificity of the kits.

### Statistical analyses

#### Age- and sex-specific probability of disease

**Extended Data Fig. 4:**
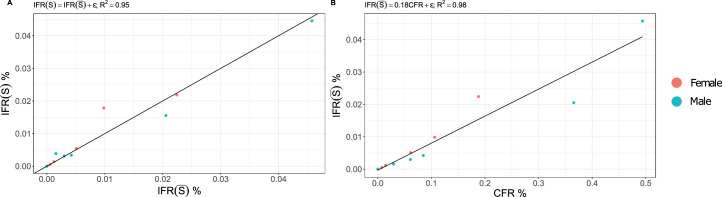
Impact of assuming equal infection risk across age and sex groups. (**a**) Infection fatality ratio (IFR) calculated using overall seroprevalence (that is, where the same attack rate is assumed for all age/sex groups) vs IFR calculated using age and sex-specific seroprevalence (that is, where we use age/sex specific attack rates from the seroprevalence study, where this is available). Red dots are females, blue dots are males. (**b**) Infection fatality ratio (IFR) calculated using overall seroprevalence *S* against Case Fatality Ratio (CFR) calculated using age and sex-specific seroprevalence. Red dots are females, blue dots are males. The lines represent the results of a linear regression.

We assumed that outside the capital subregion (Metropolitana), all individuals were susceptible before the outbreak. In the capital subregion, we assumed that 5% of individuals were seropositive before the outbreak, according to a household-based seroprevalence study in 1,000 individuals aged 5–65 years conducted in 2017 (personal communication, Paraguay Ministry of Public Health; Extended Data Fig. 4). Uncertainty on seroprevalence was accounted for by estimating the 95% CI around point estimates assuming a binomial distribution on samples tested. Seroincidence was calculated as the difference between the sensitivity-adjusted seroprevalence after the outbreak and the seroprevalence at baseline. Uncertainty from the seroprevalence estimates was propagated to the estimates on the underlying number of infections, the IFR and the probability of detection.

Given nonsignificant differences in seroprevalence estimates across sex and age groups, we assumed equal risk of exposure by age and estimated the age- and sex-specific number of infections by subregion using the attack rate and demographic data from the national census. We then estimated the probability of disease by dividing the total number of cases by age and sex strata by the estimated number of infections in the strata. We separately estimated the age- and sex-specific IFR by dividing the number of deaths in each age and sex strata by the estimated number of infections in the same strata (Extended Data Table 1). Uncertainty from the seroprevalence estimates was propagated to the estimates on the underlying number of infections, the IFR and the probability of detection.

#### Epidemic model

To characterize the chikungunya epidemic trajectory, we developed a compartmental SIR (susceptible–infected–removed) model in which the transmission rate (*β*) varies over time. We chose the SIR framework as it limits complexity and has been shown to outperform SEIR (susceptible–exposed–infected–removed) and other forms that include mosquito-specific compartments^25,26^. Weekly transmission rates were assumed to be independent and estimated as free parameters. We assumed that the generation time for CHIKV (defined as the average time interval between consecutive infections) was 2 weeks^20^.

We modeled the number of incident cases, assuming a negative binomial observation process. The likelihood of observing *c*_obs_(*t*) incident cases on week *t* given the expected number of infections *i*_exp_(*t*) for that week is given by the density of a negative binomial distribution:

$$P({c}_{\textrm{obs}}(t)|{i}_{\textrm{exp}}(t))={\mathrm{dNegBin}}({c}_{\textrm{obs}}(t),{i}_{\textrm{exp}}(t)\cdot \rho ,{\mathrm{shape}})$$

where *ρ* is the detection probability and ‘shape’ is the overdispersion parameter of the negative binomial distribution.

Within the same analytical framework using a joint likelihood approach, we incorporated the observed number of seropositive individuals in our seroprevalence study, assuming a binomial observation process. The likelihood of observing *n*_pos_(*t*) positive samples out of *n*_tot_(*t*) on week *t* given the expected proportion of susceptible individuals in the population *s*(*t*) for that week is given by the density of a binomial distribution:

$$P({n}_{\textrm{pos}}(t)|{n}_{\textrm{tot}}(t),s(t))={\mathrm{dBin}}({n}_{\textrm{tot}}(t),1-s(t))$$

The number of infectious individuals at the start of the outbreak (first week of November 2022) was fixed at 100. The detection probability is the probability that the surveillance system detected an infected individual and was estimated as a free parameter. Parameters were estimated using a Markov chain Monte Carlo (MCMC) method with a Metropolis–Hastings algorithm. We used four chains of 25,000 iterations, including a burn-in phase of 10,000 iterations and sampling with uniform noninformative priors. Effective sample sizes and R-hat values were computed for each parameter (Extended Data Table 4).

#### Estimating the impact of the vaccine

Given the absence of a traditional phase 3 trial, we do not have appropriate estimates of IXCHIQ’s efficacy against infection or disease. Therefore, we held a key stakeholder meeting during which we discussed potential parameter values with experts from Gavi, the World Health Organization and academia. Following this meeting, we developed two main scenarios: (1) a conservative scenario in which the vaccine blocked disease only with an efficacy of 75% and (2) a scenario in which the vaccine also blocked infection at the same level. We also included sensitivity analyses with a higher level of protection of 98%, consistent with other live vaccines.

To model the potential effect of a vaccine had it been available during the outbreak, we adjusted our transmission model. We assumed that the vaccine was a ‘leaky’ vaccine, such that vaccinated individuals had a probability of acquiring disease or infection despite vaccination at a predefined vaccine efficacy level. The target population was individuals aged 12 years and older, consistent with the planned initial target age group of the IXCHIQ vaccine. We initially assumed a vaccine efficacy of 0.75, meaning that 75% of vaccinated individuals were protected against disease but not against infection. We assumed a delay of 2 weeks from vaccination to acquisition of immunity^27,28^. We considered a reactive rollout campaign that started in the first week of October 2022. This date represents the moment when the Ministry of Health officially reported the outbreak. We then assumed that it takes 3 months to reach a coverage of 40% of the target population, assuming a fixed weekly rate of vaccination.

We conducted a sensitivity analysis in which we (1) varied the level of vaccination coverage and (2) allowed for different delays between the start of the outbreak and the start of campaign deployment. We also included a sensitivity analysis wherein we assumed a vaccine efficacy of 98%.

For the second main scenario, we conducted a separate analysis wherein we assumed that the vaccine blocked onward transmission at 75% efficacy. In this scenario, we assumed that vaccinated individuals had a 75% reduction in the probability of infection. Those who nevertheless became infected (that is, breakthrough infections) had the same probability of disease as infected nonvaccinated individuals.

For each scenario, we measured the number of infections, cases and deaths averted, as well as the number of doses used. In this scenario, we assumed that vaccinated individuals had a 75% reduction in the probability of infection. Those who nevertheless became infected (that is, breakthrough infections) had the same probability of disease as infected nonvaccinated individuals.

#### Software version

R v.4.4.2 (‘Pile of Leaves’) was used to run the analysis presented here.

### Ethics and inclusion statement

The project is the result of a close collaboration between the Universidad Nacional de Asunción, CENSSA and University of Cambridge. Researchers from Paraguay were integral in all stages of the research process, including study design, study implementation, data analysis and manuscript development. The first author and nine other coauthors are from Paraguay. The project was part of a knowledge exchange that was established quickly during the epidemic wherein a researcher from Cambridge (G.R.d.S.) initially worked in Paraguay to help integrate modeling efforts into the epidemic response. A researcher from Paraguay (P.E.P.-E.) then spent 2 months in Cambridge to be trained in mathematical modeling. The manuscript was developed taking existing local research into account. As there had been very little chikungunya in Paraguay historically, most of our regional understanding comes from Brazil.

### Reporting summary

Further information on research design is available in the Nature Portfolio Reporting Summary linked to this article.

## Online content

Any methods, additional references, Nature Portfolio reporting summaries, source data, extended data, supplementary information, acknowledgements, peer review information; details of author contributions and competing interests; and statements of data and code availability are available at 10.1038/s41591-025-03684-w.

## Supplementary information

Supplementary Information

Reporting Summary

## Figures and Tables

**Extended Data Fig. 3:**
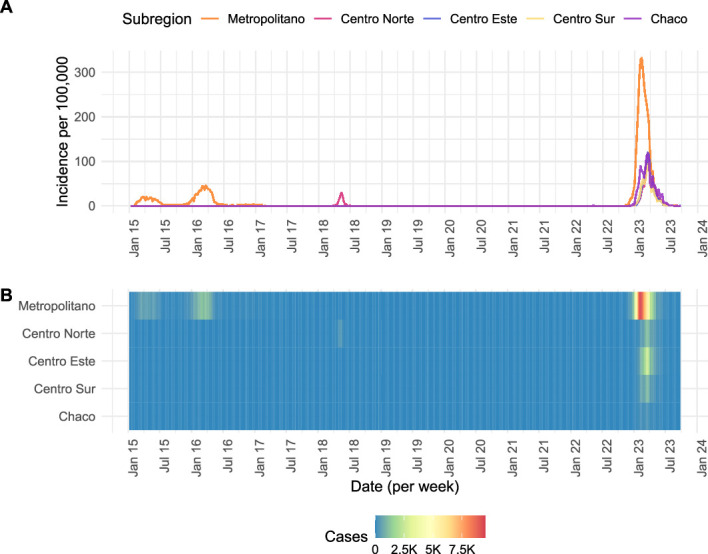
History of reported cases by subregion. (**a**) Chikungunya cases per week in Paraguay subregions between 2015–2023. (**b**) Cases reported on the same dates.

**Extended Data Table 1:**
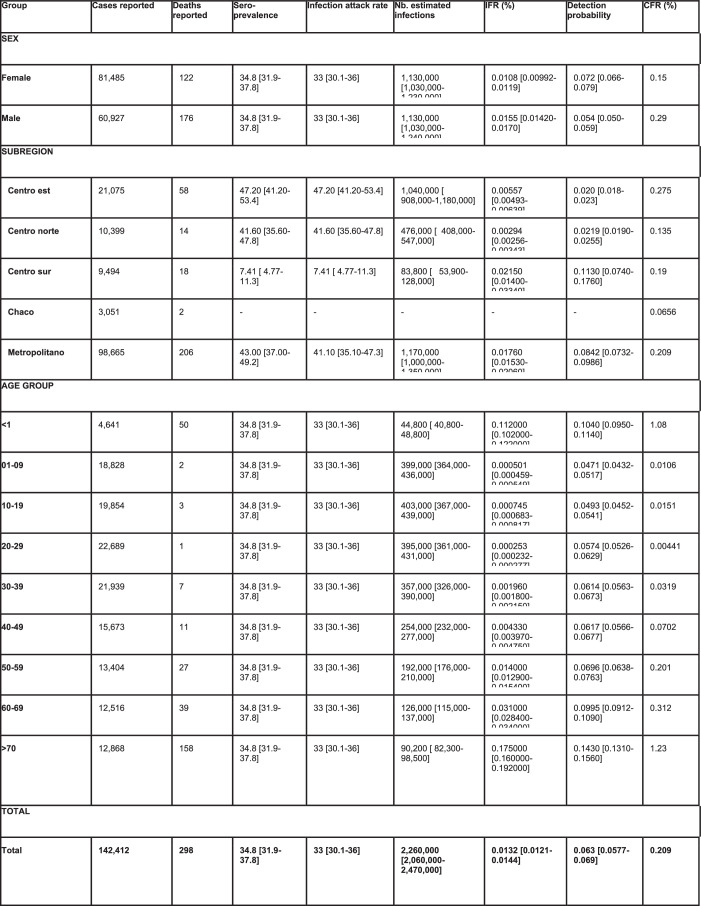
Statistics stratified by sex, subregion and age group

**Extended Data Table 2:**
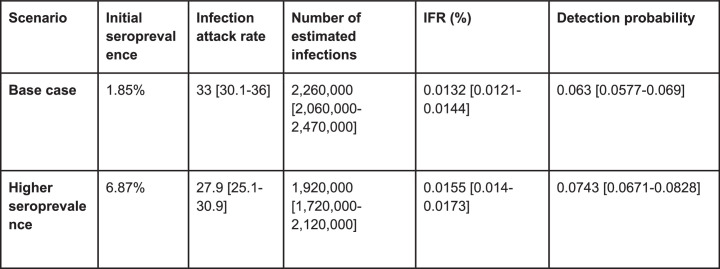
Statistics under different scenario

Sensitivity analysis comparing scenario assuming 5% initial seroprevalence in the capital and 0% elsewhere compared to 10% in the capital city and 10% elsewhere.

**Extended Data Table 3:**
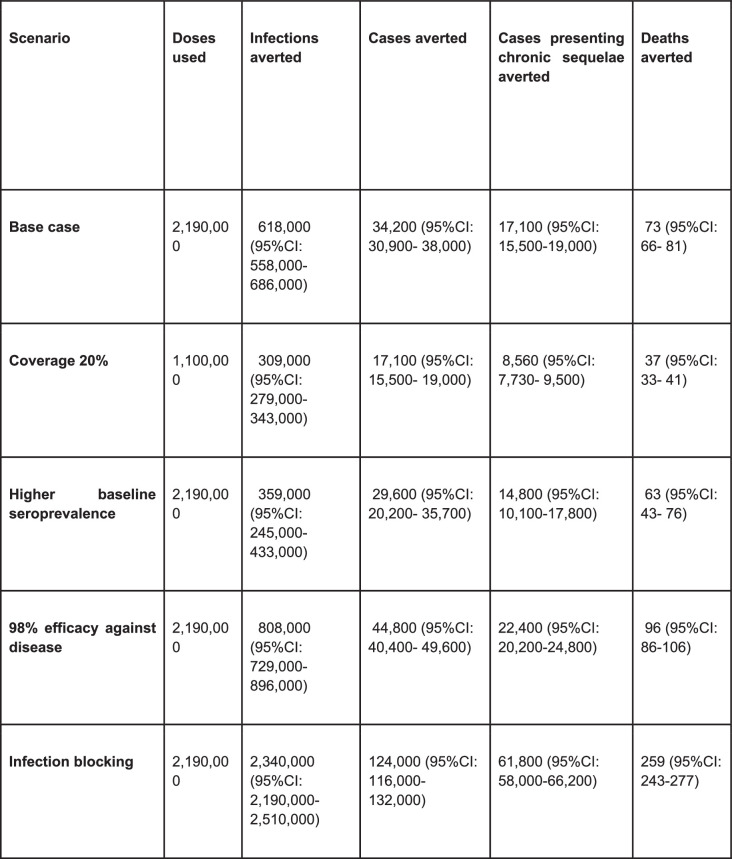
Vaccine impact under different scenarios

**Extended Data Table 4:**
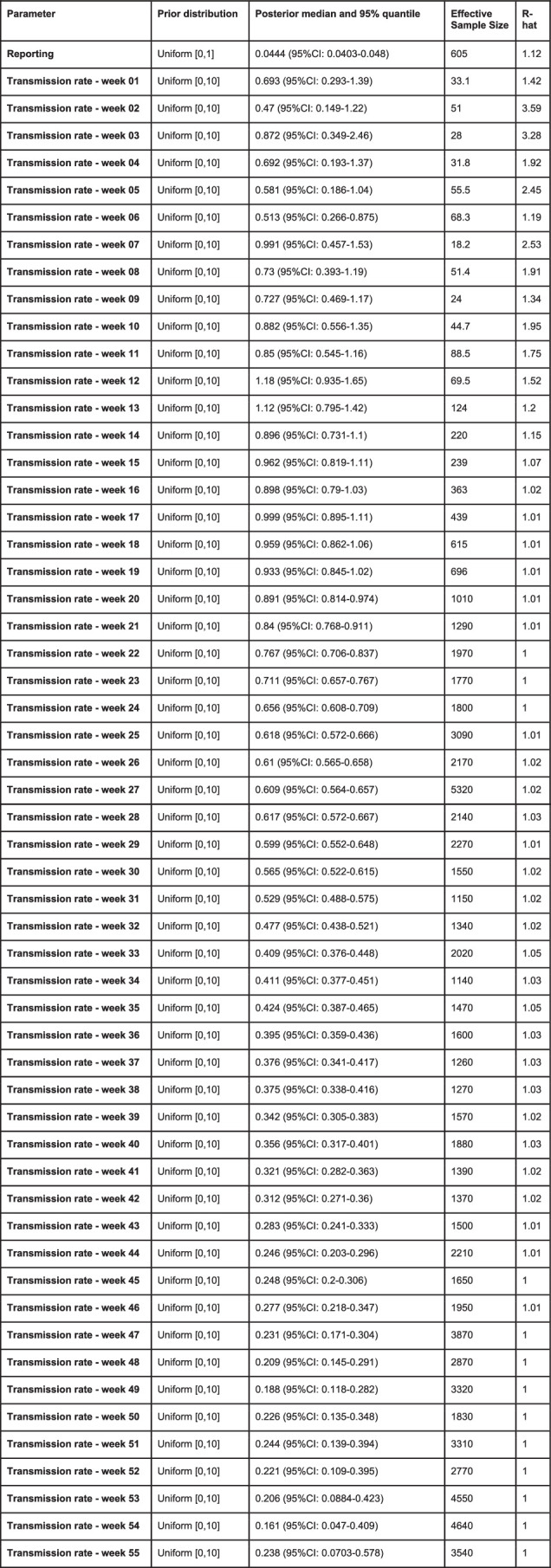
MCMC diagnostics

Prior distribution, posterior estimates, effective sample size and R-hat for all parameters estimated with the transmission model.

## Data Availability

The seroprevalence study and the case data are available in the form of anonymized line-list tables via Zenodo at 10.5281/zenodo.15263498. Data were anonymized by removing name and birth date information from the line-list data. Any external data requests can be made to P.E.P.-E. at peperez.estigarribia@gmail.com with an approximate response time of 14 days.

## Code availability

All code necessary to recreate the analyses is available via Zenodo at 10.5281/zenodo.15263498.

## References

[1] Weaver, S. C. & Forrester, N. L. Chikungunya: evolutionary history and recent epidemic spread. *Antiviral Res.* **120**, 32–39 (2015). 25979669 10.1016/j.antiviral.2015.04.016

[2] Kang, H. et al. Chikungunya seroprevalence, force of infection, and prevalence of chronic disability after infection in endemic and epidemic settings: a systematic review, meta-analysis, and modelling study. *Lancet Infect. Dis.* **24**, 488–503 (2024). 38342105 10.1016/S1473-3099(23)00810-1

[3] O’Driscoll, M., Salje, H., Chang, A. Y. & Watson, H. Arthralgia resolution rate following chikungunya virus infection. *Int. J. Infect. Dis.* **112**, 1–7 (2021). 34492392 10.1016/j.ijid.2021.08.066 PMC8627389

[4] Salje, H. & Cortés Azuero, O. The deadly potential of chikungunya virus. *Lancet Infect. Dis.* **24**, 442–444 (2024). 38342108 10.1016/S1473-3099(24)00029-X

[5] de Souza, W. M. et al. Spatiotemporal dynamics and recurrence of chikungunya virus in Brazil: an epidemiological study. *Lancet Microbe* **4**, e319–e329 (2023). 37031687 10.1016/S2666-5247(23)00033-2 PMC10281060

[6] Harris, E. FDA approves first chikungunya vaccine. *JAMA* **330**, 2241 (2023). 38019496 10.1001/jama.2023.23505

[7] Gavi, the Vaccine Alliance. More than US$ 1.8 billion in support for African vaccine manufacturing, catching up missed children and pandemic preparedness approved as Gavi Board steps up efforts to tackle backsliding and fight health emergencies. *Gavi.org* https://www.gavi.org/news/media-room/initiatives-african-vaccine-manufacturing-approved-gavi-board (2023).

[8] Desai, S. N. et al. Achievements and challenges for the use of killed oral cholera vaccines in the global stockpile era. *Hum. Vaccin. Immunother.* **13**, 579–587 (2017). 27813703 10.1080/21645515.2016.1245250 PMC5360144

[9] Kuno, G. A re-examination of the history of etiologic confusion between dengue and chikungunya. *PLoS Negl. Trop. Dis.* **9**, e0004101 (2015). 26562299 10.1371/journal.pntd.0004101 PMC4643049

[10] Yoon, I.-K. et al. High rate of subclinical chikungunya virus infection and association of neutralizing antibody with protection in a prospective cohort in the Philippines. *PLoS Negl. Trop. Dis.* **9**, e0003764 (2015). 25951202 10.1371/journal.pntd.0003764 PMC4423927

[11] Lim, J. K. et al. Seroepidemiological reconstruction of long-term chikungunya virus circulation in Burkina Faso and Gabon. *J. Infect. Dis.* **227**, 261–267 (2023). 35710849 10.1093/infdis/jiac246 PMC9833428

[12] Giovanetti, M. et al. Rapid epidemic expansion of chikungunya virus East/Central/South African lineage, Paraguay. *Emerg. Infect. Dis.* **29**, 1859–1863 (2023). 37488810 10.3201/eid2909.230523 PMC10461647

[13] Sequera, G. ¿Por qué esta gran epidemia de Chikungunya? ¿Qué paso del Dengue? *Fac. Cienc. Méd. (Asunción)* **56**, 19–24 (2023).

[14] Gutiérrez, L. A. PAHO/WHO data—weekly report. *Pan American Health Organization/World Health Organization* https://www3.paho.org/data/index.php/en/mnu-topics/chikv-en/550-chikv-weekly-en.html (2019).

[15] Yoon, I.-K. et al. Pre-existing chikungunya virus neutralizing antibodies correlate with risk of symptomatic infection and subclinical seroconversion in a Philippine cohort. *Int. J. Infect. Dis.* **95**, 167–173 (2020). 32247051 10.1016/j.ijid.2020.03.073

[16] Kumar, M. S. et al. Seroprevalence of chikungunya virus infection in India, 2017: a cross-sectional population-based serosurvey. *Lancet Microbe* **2**, e41–e47 (2021). 35544228 10.1016/S2666-5247(20)30175-0

[17] Salje, H. et al. Reconstruction of 60 years of chikungunya epidemiology in the Philippines demonstrates episodic and focal transmission. *J. Infect. Dis.* **213**, 604–610 (2016). 26410592 10.1093/infdis/jiv470 PMC4721913

[18] Schneider, M. et al. Safety and immunogenicity of a single-shot live-attenuated chikungunya vaccine: a double-blind, multicentre, randomised, placebo-controlled, phase 3 trial. *Lancet* **401**, 2138–2147 (2023). 37321235 10.1016/S0140-6736(23)00641-4 PMC10314240

[19] de Souza, W. M. et al. Chikungunya: a decade of burden in the Americas. *Lancet Reg. Health Am.* **30**, 100673 (2024). 38283942 10.1016/j.lana.2023.100673 PMC10820659

[20] Salje, H. et al. How social structures, space, and behaviors shape the spread of infectious diseases using chikungunya as a case study. *Proc. Natl Acad. Sci. USA* **113**, 13420–13425 (2016). 27821727 10.1073/pnas.1611391113 PMC5127331

[21] Hozé, N. et al. Reconstructing Mayaro virus circulation in French Guiana shows frequent spillovers. *Nat. Commun.* **11**, 2842 (2020). 32503971 10.1038/s41467-020-16516-x PMC7275077

[22] Périssé, A. R. S. et al. Zika, dengue and chikungunya population prevalence in Rio de Janeiro city, Brazil, and the importance of seroprevalence studies to estimate the real number of infected individuals. *PLoS ONE* **15**, e0243239 (2020). 33332373 10.1371/journal.pone.0243239 PMC7746276

[23] Metcalf, C. J. E. et al. Comparing the age and sex trajectories of SARS-CoV-2 morbidity and mortality with other respiratory pathogens. *R. Soc. Open Sci.* **9**, 211498 (2022). 35719888 10.1098/rsos.211498 PMC9198511

[24] Torales, M. et al. Notes from the field: chikungunya outbreak—Paraguay, 2022–2023. *MMWR Morb. Mortal. Wkly. Rep.* **72**, 636–638 (2023). 37289652 10.15585/mmwr.mm7223a5 PMC10328456

[25] Pandey, A., Mubayi, A. & Medlock, J. Comparing vector–host and SIR models for dengue transmission. *Math. Biosci.* **246**, 252–259 (2013). 24427785

[26] Andronico, A. et al. Comparing the performance of three models incorporating weather data to forecast dengue epidemics in Reunion Island, 2018–2019. *J. Infect. Dis.* **229**, 10–18 (2024). 37988167 10.1093/infdis/jiad468 PMC10786251

[27] Chen, L. H., Fritzer, A., Hochreiter, R., Dubischar, K. & Meyer, S. From bench to clinic: the development of VLA1553/IXCHIQ, a live-attenuated chikungunya vaccine. *J. Travel Med.* **31**, taae123 (2024). 39255380 10.1093/jtm/taae123 PMC11497415

[28] McMahon, R. et al. A randomized, double-blinded phase 3 study to demonstrate lot-to-lot consistency and to confirm immunogenicity and safety of the live-attenuated chikungunya virus vaccine candidate VLA1553 in healthy adults. *J. Travel Med.* **31**, taad156 (2024). 38091981 10.1093/jtm/taad156 PMC10911060

