# Supplementary material for: Modeling the impact of vaccine campaigns on the epidemic transmission dynamics of chikungunya virus outbreaks
Source: Nat Med. 2025 May 1;31(7):2335–41. doi: 10.1038/s41591-025-03684-w (PMC12283388; doi:10.1038/s41591-025-03684-w)
Supplement: Supplementary file 1 — Supplementary Information [file 41591_2025_3684_MOESM1_ESM.pdf]

nature medicine

Supplementary information https://doi.org/10.1038/s41591-025-03684-w

Modeling the impact of vaccine campaigns on the epidemic transmission dynamics of chikungunya virus outbreaks

In the format provided by the authors and unedited

Supplementary Information

SIRV ODE system

The outbreak was characterised using an SIR compartmental model. To estimate the potential impact of a vaccine it was estimated adding vaccinated compartments to the SIR system. We modelled vaccine efficacy (VE) as being a leaky vaccine, with all recipients of the doses receiving partial protection. The set of equations is as follow:

$$\frac{dS_a}{dt} = -\triangle_{SIa} - \triangle_{SVa}$$

$$\frac{dV_a}{dt} = \triangle_{SVa} - \triangle_{VIVa}$$

$$\frac{I_a}{dt} = \triangle_{SIa} - \triangle_{IRa}$$

$$\frac{dIV_a}{dt} = \triangle_{VIVa} - \triangle_{IVRVa}$$

$$\frac{dR_a}{dt} = \triangle_{IRa} - \triangle_{RRVa}$$

$$\frac{dRV_a}{dt} = \triangle_{IVRVa} + \triangle_{RRVa}$$

With :

- $\triangle_{SIa} = \beta_t * \frac{I_{tot}}{N} * S_a$
- $\triangle_{VIVa} = \beta_t * (1 - v_{ei}) * \frac{I_{tot}}{N} * V_a$
- $\triangle_{IRa} = \sigma * I_a$
- $\triangle_{IVRVa} = \sigma * IV_a$
- $\triangle_{SVa}$ and $\triangle_{RRVa}$ being dictated by the daily rates of vaccination from the campaign
- $I_{tot} = \sum_a (I_a + IV_a)$, the total number of infected individuals.
- $N = \sum_a (S_a + V_a + I_a + IV_a + R_a + RV_a)$, the total population size
- v_{ei} the vaccine-induced protection against infection
- $1/\sigma$ is the mean duration of infectiousness, fixed at 15 days [20] .
- β_t is the transmission rate estimated at time t
