# Supplementary material for: Modeling the impact of vaccine campaigns on the epidemic transmission dynamics of chikungunya virus outbreaks
Source: Nat Med. 2025 May 1;31(7):2335–41. doi: 10.1038/s41591-025-03684-w (PMC12283388; doi:10.1038/s41591-025-03684-w)
Supplement: Supplementary file 2 — Reporting Summary [file 41591_2025_3684_MOESM2_ESM.pdf]

nature portfolio

Corresponding author(s): Henrik Salje

Last updated by author(s): 03/03/2025

Reporting Summary

Nature Portfolio wishes to improve the reproducibility of the work that we publish. This form provides structure for consistency and transparency in reporting. For further information on Nature Portfolio policies, see our Editorial Policies and the Editorial Policy Checklist.

Statistics

For all statistical analyses, confirm that the following items are present in the figure legend, table legend, main text, or Methods section.

n/a	Confirmed	
☐	☒	The exact sample size (n) for each experimental group/condition, given as a discrete number and unit of measurement
☒	☐	A statement on whether measurements were taken from distinct samples or whether the same sample was measured repeatedly
☒	☐	The statistical test(s) used AND whether they are one- or two-sided<br>*Only common tests should be described solely by name; describe more complex techniques in the Methods section.*
☒	☐	A description of all covariates tested
☒	☐	A description of any assumptions or corrections, such as tests of normality and adjustment for multiple comparisons
☐	☒	A full description of the statistical parameters including central tendency (e.g. means) or other basic estimates (e.g. regression coefficient) AND variation (e.g. standard deviation) or associated estimates of uncertainty (e.g. confidence intervals)
☐	☒	For null hypothesis testing, the test statistic (e.g. F, t, r) with confidence intervals, effect sizes, degrees of freedom and P value noted<br>*Give P values as exact values whenever suitable.*
☐	☒	For Bayesian analysis, information on the choice of priors and Markov chain Monte Carlo settings
☒	☐	For hierarchical and complex designs, identification of the appropriate level for tests and full reporting of outcomes
☐	☒	Estimates of effect sizes (e.g. Cohen's d, Pearson's r), indicating how they were calculated

Our web collection on statistics for biologists contains articles on many of the points above.

Software and code

Policy information about availability of computer code

Data collection	No software was used for data collection
Data analysis	All analyses were done using R v4.4.2 - a Metropolis-Hastings algorithm was implemented.

For manuscripts utilizing custom algorithms or software that are central to the research but not yet described in published literature, software must be made available to editors and reviewers. We strongly encourage code deposition in a community repository (e.g. GitHub). See the Nature Portfolio guidelines for submitting code & software for further information.

Data

Policy information about availability of data

All manuscripts must include a data availability statement. This statement should provide the following information, where applicable:

- Accession codes, unique identifiers, or web links for publicly available datasets
- A description of any restrictions on data availability
- For clinical datasets or third party data, please ensure that the statement adheres to our policy

The seroprevalence study as well as the case data are available in the form of anonymised line list tables on : https://github.com/Pastor-E-Perez-Estigarribia/ePY .

Research involving human participants, their data, or biological material

Policy information about studies with human participants or human data. See also policy information about sex, gender (identity/presentation), and sexual orientation and race, ethnicity and racism.

Reporting on sex and gender	Our analysis investigates differences in exposure risk and outcome severity by sex. We did not investigate differences by gender.
Reporting on race, ethnicity, or other socially relevant groupings	N/A
Population characteristics	Our analysis investigates differences in exposure risk and outcome severity by age at exposure.
Recruitment	We analysed deidentified serum samples from blood donors across the country of Paraguay.This creates a selection bias as we sampled the popoulation with access to blood centers and willingness to donate blood.
Ethics oversight	The seroprevalence study was conducted as part of the Paraguay Ministry of Health PublicHealth Emergency Response and, therefore, did not require ethical approval.

Note that full information on the approval of the study protocol must also be provided in the manuscript.

Field-specific reporting

Please select the one below that is the best fit for your research. If you are not sure, read the appropriate sections before making your selection.

☒ Life sciences ☐ Behavioural & social sciences ☐ Ecological, evolutionary & environmental sciences

For a reference copy of the document with all sections, see nature.com/documents/nr-reporting-summary-flat.pdf

Life sciences study design

All studies must disclose on these points even when the disclosure is negative.

Sample size	Sample size for the seroprevalence study was based on available funding at the time. 1,000 samples is enough to give a seroprevalece estimate with 95% confidence interval at +/- 3% .
Data exclusions	No data was excluded from the study.
Replication	Replication was not necessary as we used commercially available kits and account for its sensitivity and specificity in our seroprevalence estimate.
Randomization	Randomization was not applicable in our study as it was not a clinical trial and there was no intervention/control arm to assign people to.
Blinding	Blinding was not applicable in our study as it was not a clinical trial and there was no intervention to blind participants/clinicians from.

Reporting for specific materials, systems and methods

We require information from authors about some types of materials, experimental systems and methods used in many studies. Here, indicate whether each material, system or method listed is relevant to your study. If you are not sure if a list item applies to your research, read the appropriate section before selecting a response.

Materials & experimental systems

n/a	Involved in the study	
☒	☐	Antibodies
☒	☐	Eukaryotic cell lines
☒	☐	Palaeontology and archaeology
☒	☐	Animals and other organisms
☐	☒	Clinical data
☒	☐	Dual use research of concern
☒	☐	Plants

Methods

n/a	Involved in the study	
☒	☐	ChIP-seq
☒	☐	Flow cytometry
☒	☐	MRI-based neuroimaging

Clinical data

Policy information about clinical studies

All manuscripts should comply with the ICMJE guidelines for publication of clinical research and a completed CONSORT checklist must be included with all submissions.

Clinical trial registration	na
Study protocol	na
Data collection	Epidemiological data was extracted from the national surveillance database. Deidentified blood samples were collected from the national blood bank.
Outcomes	na

Plants

Seed stocks	na
Novel plant genotypes	na
Authentication	na
